# Assessing impact of passive virtual reality exposure intervention on physiological parameters in caregivers of individuals suffering from schizophrenia: A pilot study protocol

**DOI:** 10.1192/j.eurpsy.2025.407

**Published:** 2025-08-26

**Authors:** R. Verma, V. Patil, I. Dhyani, S. Karna

**Affiliations:** 1 Psychiatry, All India Institute of Medical Sciences, New Delhi, India

## Abstract

**Introduction:**

Caregiving to individuals with schizophrenia is intensive, complex, and long lasting. In developing countries, the primary caregivers are family members who rarely receive adequate preparation for their role. The diversity and intensity of caregiving roles also may result in caregiver strain and burden. Interaction with nature even in form of hearing sounds have been found to reduce stress markers. Immersive Virtual Environments (IVEs) can result in restorative effects such as increased positive affect, decreased negative affect, and decreased stress.

**Objectives:**

To assess and compare the effect of combined use of nature-based VR and nature-based sounds to nature-based sounds only on physiological parameters (heart rate, respiratory rate, oxygen saturation, systolic and diastolic blood pressure) in caregivers of individuals having schizophrenia.

**Methods:**

Sixty caregivers (aged more than 18 years) of inpatients with schizophrenia as per Diagnostic & Statistical Manual (DSM-5) will be recruited with consecutive sampling. Caregivers should have been staying with patient for at least previous 1 year and have been staying in the ward for previous 7 days for at least 12 hours/day. Individuals with hearing or visual deficits, history of having received treatment for mental illness/epilepsy, or taking sleeping pills/sedatives/hypnotics/cough syrup currently would be excluded. Paid caregiver and those not willing to provide written informed consent would be excluded from the study. Data collection tools will include a semi-structured proforma for mentioning the socio demographic details, checklist for observing physiological parameters (Heart Rate, Respiratory Rate, Oxygen Saturation, and Blood Pressure), Perceived Stress Scale (PSS), Pittsburg Sleep Quality Index (PSQI), Satisfaction with Life Scale (SwLS), Subjective stress on Visual analogue scale (VAS), User Satisfaction Evaluation Questionnaire (USEQ) and VR Experience Questionnaire (VEQ). Participants would be divided randomly into two groups with group 1 (n=30) individuals receiving only nature-based sounds intervention and those in group 2 (n=30) would receive combined nature-based VR and nature-based sounds intervention.

**Results:**

Primary outcome will be change in physiological parameters using repeated measures ANOVA. Additional outcomes like acceptability and feasibility of the intervention will also be reported as descriptive data. Pearson’s correlation will be done between scale scores and change in physiological parameters in different groups based upon nature of intervention, illness based or gender based.

**Conclusions:**

The study provides insight into the utility of upcoming VR interventions to cater the needs of caregivers to individuals with schizophrenia in moderating the stress levels.

**Disclosure of Interest:**

None Declared

